# Metabolic encephalopathy secondary to diabetic ketoacidosis: a case report

**DOI:** 10.1186/s12902-019-0398-8

**Published:** 2019-07-02

**Authors:** Maria Tomkins, Richard McCormack, Karen O’Connell, Amar Agha, Áine Merwick

**Affiliations:** 1 Department of Diabetes and Endocrinology, Beaumont Hospital, Dublin, Ireland; 2 Department of Rehabilitation, National Rehabilitation Hospital, Dublin, Ireland; 3 Department of Neurology, Beaumont Hospital, Dublin, Ireland; 4 Department of Diabetes and Endocrinology, Beaumont Hospital, Dublin, Ireland

**Keywords:** Diabetes mellitus, Metabolic encephalopathy, Ketoacidosis, Diabetic brain injury, Type 1 diabetes

## Abstract

**Introduction:**

Metabolic encephalopathy is a rare but potentially devastating complication of diabetic ketoacidosis (DKA). This case highlights the dramatic cognitive decline of a young man due to metabolic encephalopathy complicating DKA. The aims of this case report are to highlight metabolic encephalopathy as a complication of DKA and to explore the current research in diabetic related brain injury. The importance of investigation and treatment of reversible causes of encephalopathy is also demonstrated.

**Case presentation:**

A 35-year-old man with a background of type 1 diabetes mellitus (T1DM) and relapsing remitting multiple sclerosis (RRMS) presented to the emergency department (ED) in a confused and agitated state. Prior to admission he worked as a caretaker in a school, smoked ten cigarettes per day, took excess alcohol and smoked cannabis twice per week.

Following initial investigations, he was found to be in DKA. Despite timely and appropriate management his neurological symptoms and behavioural disturbance persisted. Neuroimaging revealed temporal lobe abnormalities consistent with an encephalopathic process. The patient underwent extensive investigation looking for evidence of autoimmune, infective, metabolic, toxic and paraneoplastic encephalopathy, with no obvious cause demonstrated.

Due to persistent radiological abnormalities a temporal lobe biopsy was performed which showed marked astrocytic gliosis without evidence of vasculitis, inflammation, infarction or neoplasia. A diagnosis of metabolic encephalopathy secondary to DKA was reached. The patient’s cognitive function remained impaired up to 18 months post presentation and he ultimately required residential care.

**Conclusions:**

Metabolic encephalopathy has been associated with acute insults such as DKA, but importantly, the risk of cerebral injury is also related to chronic hyperglycaemia. Mechanisms of cerebral injury in diabetes mellitus continue to be investigated. DKA poses a serious and significant neurological risk to patients with diabetes mellitus. To our knowledge this is the second case report describing this acute complication.

## Background

Diabetic ketoacidosis is a frequent complication of type 1 diabetes, with the UK National Diabetes Audit quoting a crude incidence rate of 3.6% among patients with type 1 diabetes [1]. Risk factors for DKA include younger age, poor glycaemic control, lower socioeconomic status and depression/psychiatric illness. Precipitants of DKA include omission of or inadequate insulin, infection, cardiovascular disease such as stroke/acute coronary syndrome, acute pancreatitis and certain medications such as steroids, thiazide diuretics and sodium-glucose-co-transporter 2 (SGLT2) inhibitors. Physiological stress from surgery, pregnancy or trauma has the potential to initiate a DKA due to increased release of counter-regulatory hormones [1].

Metabolic encephalopathy refers to an alteration in consciousness due to impaired cerebral metabolism causing diffuse or global brain dysfunction in the absence of primary structural dysfunction [2]. Chemical imbalance occurs in the setting of hypoxia or due to systemic organ failure (hepatic failure, renal failure, pancreatic failure) and electrolyte imbalance (hypercalcaemia, hypoglycaemia, hyponatraemia) resulting in toxic neurological injury [2]. Varying presentations occur and may indicate the underlying cause, for example patients with hepatic encephalopathy may demonstrate asterixis, however in general metabolic encephalopathy presents as global cerebral dysfunction with fluctuating consciousness in the absence of focal neurological signs [3, 4]. It is a very rare but potentially devastating complication of diabetic ketoacidosis (DKA). Hitherto, only one case has been reported of this complication in which the patient showed a gradual recovery with residual speech disturbance and irritability [5].
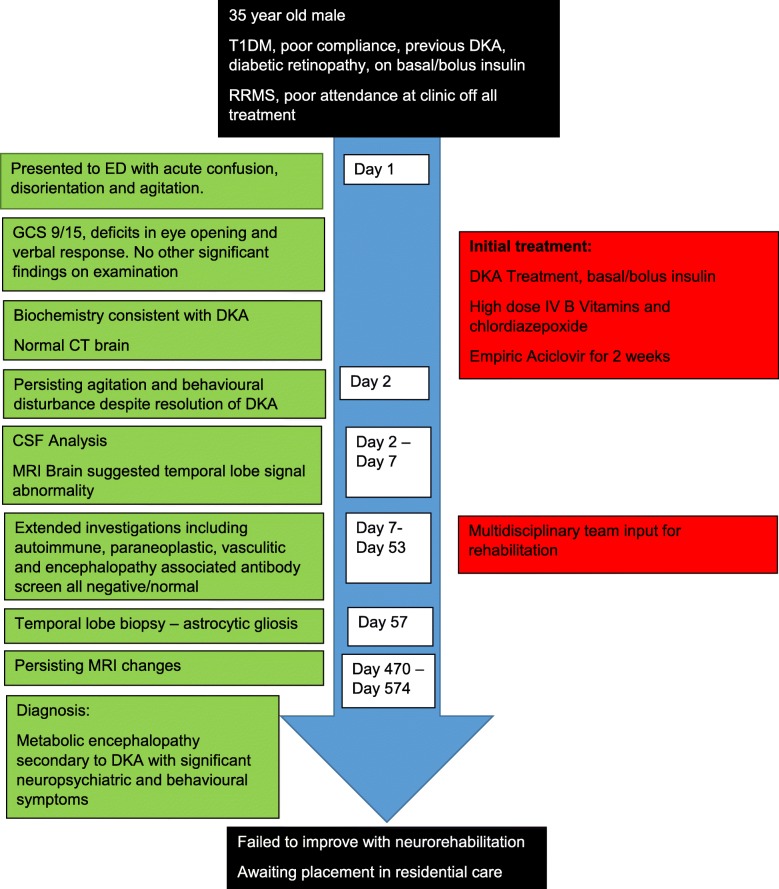


In this paper we describe a case of a patient with persisting neurological injury secondary to metabolic encephalopathy associated with DKA. The aims of this case report are to highlight metabolic encephalopathy as a complication of DKA and to explore the current research in diabetic related brain injury. The importance of investigation and treatment of reversible causes of encephalopathy is also demonstrated, as this is a diagnosis of exclusion.

## Case presentation

A 35-year-old man with T1DM presented to ED, having been found in an acutely confused state at home. Having not left his bedroom for 2 days, his co-habitants alerted emergency services who forced entry to his bedroom and found him in an unkempt, confused state. On arrival he was agitated, confused, unkempt and uncommunicative. The majority of the clinical history was provided by his parents who had seen him well 2 days previously. They described an independent 35-year-old man who had no complaints in the days leading up to his admission. They described poor engagement with medical services regarding his diabetes and multiple sclerosis. His social and recreational history was provided by the family, who were aware of his occasional illicit drug use, excessive alcohol intake and smoking status. Additional information regarding his past medical interventions and treatments was available in his medical record.

**Fig. 1:**
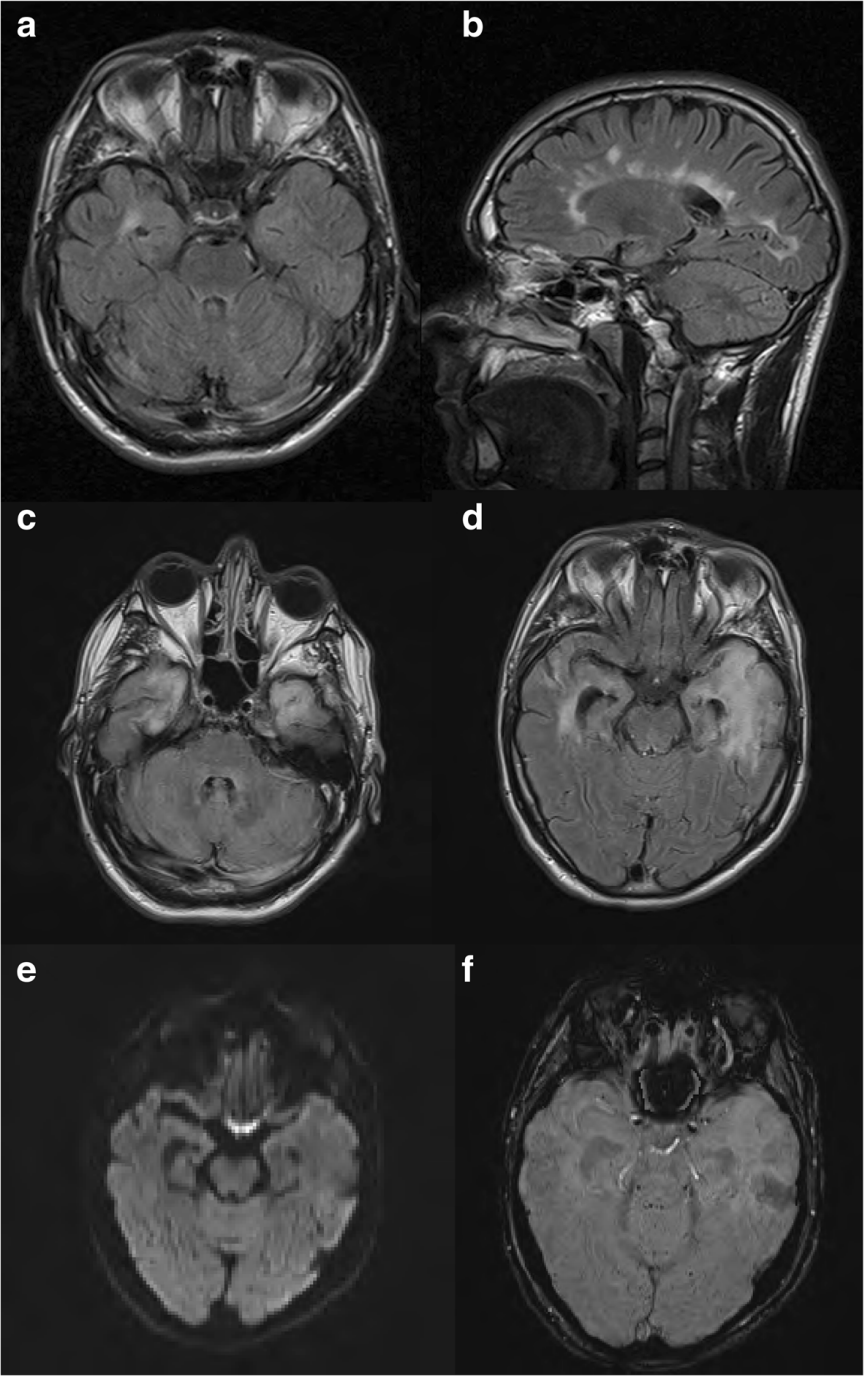
Axial FLAIR (**a**) and saggital FLAIR (**b**) sequences from last surveillance MRI for his MS taken 2 months prior to presentation shows stable periventricular white matter lesions with evidence of an isolated demyelinating plaque in his right temporal horn. Axial FLAIR (**c** and **d**) sequence at presentation shows high signal abnormality in both temporal lobes extending to the insular cortex. Axial diffusion weighted imaging (**e**) shows no restricted diffusion and susceptibility weighted imaging (**f**) shows no area of focal haemorrhage

His medical background was significant for T1DM, diagnosed at age nine. He was taking basal/bolus insulin. His diabetes was complicated by background diabetic retinopathy. He poorly engaged with diabetes services and had not attended his diabetes clinic appointments for two years prior to presentation, solely attending his general practitioner when repeat insulin prescriptions were required. He had a history of poor glycaemic control with HbA1c ranging from 67 to 99 mmol/mol (8.3 to 11.2%) over the previous ten years, one previous DKA eleven years prior which was attributed to excess alcohol intake and omission of insulin and one previous hypoglycaemic seizure following incorrect self-administration of insulin. Relapsing Remitting Multiple Sclerosis (RRMS) was diagnosed at age 26, and he was an infrequent attender of the neurology clinic, having previously been prescribed interferon beta but had self-discontinued using 5 years previous. His multiple sclerosis had been clinically and radiographically stable; with most recent MRI brain performed 2 months prior to his presentation (Fig. 1). He had been suffering with mild to moderate depression for four years prior to admission and was taking escitalopram. He also had experienced a suspected seizure six months prior to his presentation however, unfortunately, he failed to attend for investigation of this episode.

There was no significant family history. He worked as a caretaker in a school, was living independently and smoked ten cigarettes per day. He also was known to take excess alcohol, and smoked cannabis twice per week.

On examination his vital signs were normal however his Glasgow Coma Scale (GCS) was 9 (eye opening 3, verbal response 1, motor response 5). Cardiovascular, respiratory and abdominal exams were normal. Focused neurological examination including cranial nerves, fundoscopy, gait, tone and reflexes did not show a focal deficit but was limited especially in higher cortical function assessment by inability of the patient to co-operate with examination.

### Investigations

Biochemistry was consistent with DKA (pH 7.17, blood ketones 8 mmol/L and blood glucose 26 mmol/L). Alcohol levels were undetectable, urine and serum toxicology screens were negative and there was no clinical, biochemical or radiological evidence of infection. Full blood count, urea and electrolytes, liver function, and C-reactive protein were normal. Thyroid function, iron, ferritin and B12 were normal, however he was folate deficient (2.3μg/L). HbA1c was 70 mmol/mol (8.5%). Computed tomography (CT) imaging of the brain showed no acute pathology.

Cerebrospinal fluid (CSF) analysis on the second day of admission revealed an elevated protein at 61 mg/dl with normal glucose 6.3 mol/L, erythrocytes 86u/L and leucocytes 1/uL. CSF Viral PCR for Herpes Simplex Virus 1 + 2, Varicella Zoster Virus, Enterovirus, Human Herpes Virus 6, Epstein Barr Virus DNA, John Cunningham Virus (JCV) DNA and cytomegalovirus were all negative. CSF cytology showed no evidence of abnormal or malignant cells. Serum and CSF gram stain and culture were both negative. Serum Treponema pallidum, HBV, HCV and HIV 1 + 2 were negative.

Connective tissue disease screen including antibodies to anti-nuclear factor, double stranded DNA, RNP, anti-phospholipid, Smith, Ro and La were all negative. Beta-2 glycoprotein and anticardiolipin IgG and IgM were normal. Immunoglobulins G, A and M and serum protein electrophoresis were normal.

Serum paraneoplastic antibodies were negative. Extensive immunological screen of anti-GFAP, anti = GAD65, anti-MOG, anti-GABA_B_ receptor, anti-AMPA I + II, Aquaporin 4 NMO, Anti TTG antibodies were negative. Immunofluorescence and immunoblot did not reveal evidence of Anti-Yo, Anti-Hu, Anti- Ri, Anti Ma1, Anti Ma2, anti-cv2/CRMP5, Amphiphysin, Sox-1, Zic-4, anti-Tr antibodies. Anti-VGKC, anti-NMDA receptor and anti-TPO antibodies were negative. Carnitine, homocysteine, vitamin D and ammonia levels were normal. Mitochondrial POLG genetics were negative. Anti-glutamic acid decarboxylase levels were negative/within normal range.

An Electroencephalogram (EEG) was attempted repeatedly, but unfortunately was abandoned on a number of occasions, due to severe agitation. When obtained three weeks after presentation, EEG showed global cerebral dysfunction without definite epileptiform features and no electroencephalographic seizures were detected.

MRI brain scan showed new diffuse high signal changes in both temporal lobes and hippocampi (Fig. 1c-f). He also had a number of subcortical and periventricular demyelinating plaques, that were stable in number and size compared with his most recent RRMS surveillance MRI (Fig. 1a-b). Follow-up imaging at 6 weeks showed progression of these changes with high signal now extending into the insular cortex bilaterally.

**Fig. 2:**
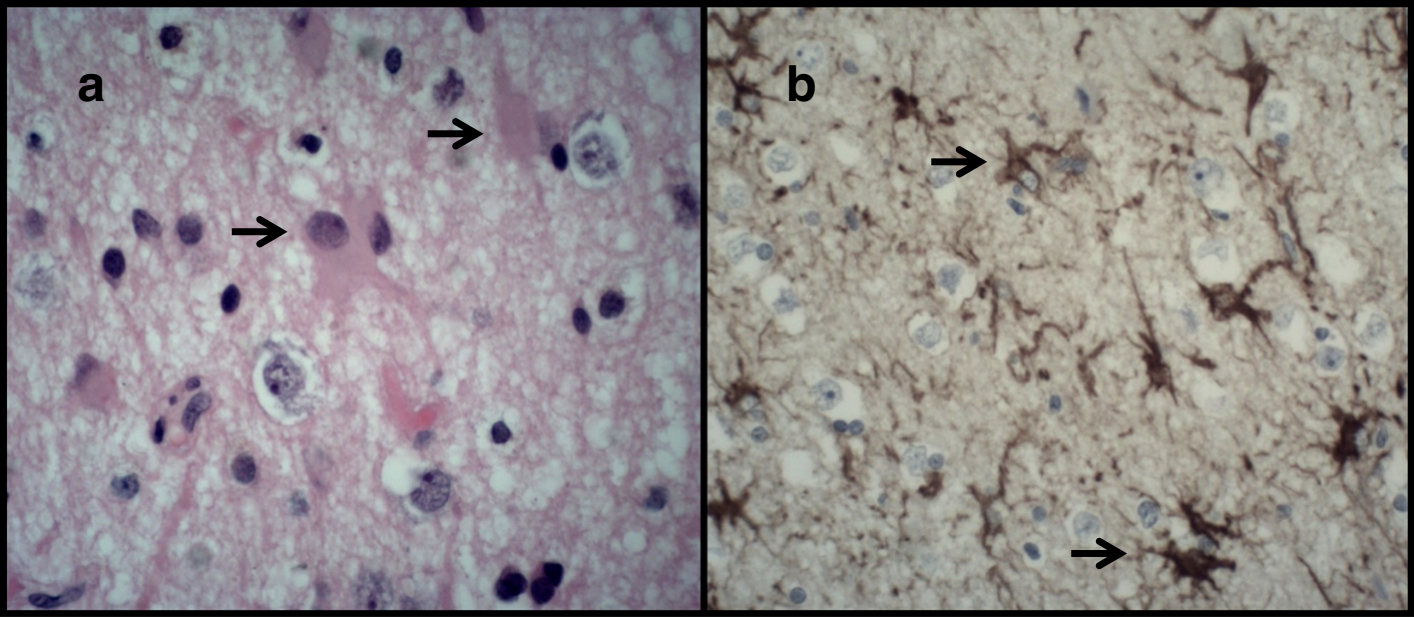
Astrocytic gliosis. (**a**) H&E and (**b**) glial fibrillary acid protein (GFAP) stained sections demonstrate marked astrocytic gliosis characterised by an evenly dispersed proliferation of large reactive astrocytes with abundant eosinophilic cytoplasm and branching processes (arrows)

Due to these progressive radiological changes and lack of clinical improvement the patient underwent a temporal lobe biopsy which showed marked astrocytic gliosis, without evidence of vasculitis, diffuse parenchymal inflammation, infarction or neoplasia (Fig. 2a-b). Immunostaining for HSV1, HSV2 and SV40 (JCV marker) were all negative.

### Timeline

### Differential diagnosis

The precipitant of DKA in this case is unclear given his absent period prior to admission, and may have been multifactorial. We postulate that it may be due to a combination of omission of insulin with or without alcohol misuse, as this was his previous precipitant. Starvation and alcohol excess may have also contributed to ketoacidosis.

There are multiple differentials of encephalopathy in this case. As the patient was not seen for two days prior to presentation he may have had an unwitnessed traumatic brain injury however this was not apparent on imaging. Toxic brain injury was also considered however the radiological findings were inconsistent with this and toxicology screen was negative. Wernicke’s encephalopathy was considered given his previous alcohol history, however there was no improvement with high dose B vitamin supplementation and the clinical and imaging features would not be in keeping with that diagnosis. Infectious aetiology should always be considered in patients presenting with encephalopathy. Indeed, the finding of high signal within the temporal lobes extending to the insula would fit with a viral encephalitis, such as that caused by HSV. However, the lack of diffusion restriction and microhaemorrhages on his imaging, lack of brain biopsy features as well as repeatedly negative CSF viral PCR and normal leucocyte count make this diagnosis unlikely. Other rarer causes of encephalopathy including autoimmune, vasculitic and paraneoplastic disease were also explored and ruled out. Sub-clinical seizure activity may have caused his persistent behavioural disturbance in the acute phase but unfortunately the patient was initially unable to cooperate with EEG testing and there were no features on repeated clinical examination by a neurologist of overt seizures. Nutritional deficiency such B vitamin / folate deficiency could have also played a role, the patient had mild folate deficiency which would not typically cause such a devastating neurological injury, however may have contributed to susceptibility to brain injury.

Cerebral oedema due to diabetic encephalopathy can cause brain injury however there was no clinical or radiographical evidence of this. Therefore, we feel that metabolic encephalopathy due to DKA is the most likely diagnosis in this case.

The differential of metabolic encephalopathy secondary to DKA was raised approximately one week into the clinical admission following negative viral PCR and lack of improvement with thiamine and chlordiazepoxide. Initially the most likely diagnosis was a viral or toxic encephalopathy, however with no clinical improvement to treatment a wider differential was considered. A comprehensive suite of investigations ensued to undercover the aetiology of the patient’s behavioural disturbance, however neither imaging, laboratory or pathological specimens led to a definite cause. Following multidisciplinary discussion, the diagnosis of metabolic encephalopathy secondary to DKA was reached.

### Treatment and clinical course

He was commenced on a DKA management protocol which consisted of aggressive intravenous fluid resuscitation, intravenous continuous insulin infusion and intravenous and oral replacement of potassium, phosphate and magnesium. DKA management protocol was continued for forty-eight hours, at which point all biochemical markers were within normal limits and the patient was transitioned to basal-bolus insulin regimen. In addition, the patient was also given intravenous high dose B vitamins and a reducing regimen of chlordiazepoxide over one week. He received folic acid supplementation 5 mg daily for two months. Subsequent treatment was largely supportive. Empiric antiviral therapy was given for HSV (later HSV PCR came back as negative, although two-week course complete). He underwent intensive rehabilitation involving a multidisciplinary team of occupational therapy, physiotherapy, social care and neuropsychology.

Subsequent imaging at 3, 4 and 6 months post presentation demonstrated persistent but stable high-signal changes in the temporal lobes bilaterally.

Unfortunately following temporal lobe biopsy the patient experienced generalized tonic clonic seizures which were difficult to control, requiring three antiepileptic agents. EEG was performed at this point, ten weeks into his admission following temporal lobe biopsy. The findings were in keeping with an encephalopathic state with a highly epileptogenic focus in the left frontocentral region.

He remained relatively unchanged for the next 18 months with persistent severe global cognitive impairment with most marked deficits in attention, short-term memory and ability to learn new information. He had ongoing erratic control of his blood sugars, attributable to inconsistent oral intake. He continued to have seizures with generalised tonic-clonic events occurring approximately once per month. Behavioural issues remained a problem and he required constant supervision, ultimately requiring long term residential care.

## Discussion and conclusions

Regarding the strengths of this case report, it is clear that the patient was extensively investigated to ensure possible reversible causes were outruled. Limitations of this case report include the lack of EEG in the acute phase due to patient agitation.

From review of the literature, brain injury as a result of DKA has been well described in the paediatric population however the mechanisms of brain injury remain unclear. This uncertainty is mostly due to the myriad of metabolic disturbances which occur during DKA [6]. There is a paucity of cases describing metabolic encephalopathy secondary to DKA in the adult population. Miras et al. described a very similar case in a 44 year old man [5]. Similarly, he was found collapsed and had not been contactable for 3 days prior to his admission. Interestingly, he also had a history of alcohol excess and poorly controlled type 1 diabetes with recurrent severe hypoglycaemic episodes. In this case the patient had significant behavioural disturbance with aggression and confusion. Neuroimaging was entirely normal, CSF examination was bland and EEG showed diffuse slowing consistent with encephalopathy. Over the following six months this patient gradually improved with neuropsychiatric rehabilitation however had residual irritability and slow speech [5].

DKA has been shown to induce brain injury however the exact pathogenesis of this injury is debated. In paediatric cases subclinical cerebral oedema is common, with frank cerebral oedema occurring in 0.5–1% of children with DKA [7]. Historically cerebral oedema secondary to DKA was thought to be due to overaggressive fluid resuscitation and loss of brain osmotic homeostasis, however recently this theory has been challenged. Cerebral hypoperfusion followed by reperfusion injury correlates with the spectrum of cytotoxic and vasogenic oedema seen in patients with cerebral oedema due to DKA and is now hypothesized to be the mechanism of brain injury in DKA [7]. However, cerebral oedema in the setting of DKA or Hyperglycaemic hyperosmolar syndrome (HHS) in adults is exceedingly rare, with a United States large population-based study revealing a 0.03% incidence rate [8]. In the case outlined above there was no evidence of cerebral oedema clinically or radiologically, therefore other mechanisms of brain injury must be at play.

Interestingly Jessup et al., studied a cohort of young patients with new onset type 1 diabetes and found that patients who presented with DKA scored lower on visual cognitive tasks when compared to age-matched patients without DKA. Cognitive disparity between the two groups remained 8–12 weeks post discharge. The authors suggest that metabolic dysregulation during DKA mediates neuroinflammation and cerebral oxidative stress causing a neuronal injury [9]. This is further supported histologically, where examination of brain tissue from patients with DKA and cerebral oedema showed evidence of oxidative stress, with increased products of oxidative damage present in vulnerable brain areas compared to healthy controls [10]. Hoffman et al., also showed that both acute and chronic metabolic dysregulation in T1DM can affect brain function, promoting neuroinflammation, cerebral insulin resistance and reduced insulin signalling, culminating in increased oxidative and inflammatory cerebral stress [10]. Chronic hyperglycaemia, ketoacidosis and dehydration with superimposed acute insults, in the form of DKA, can subsequently cause diabetic encephalopathy [10]. The long-term erratic glycaemic control in the case outlined above may therefore have also played a role in the patient’s cognitive decline.

Recent research has indeed demonstrated chronic altered brain metabolism and signalling as a cause of diabetic brain dysfunction. Analysing brain metabolites with magnetic resonance spectroscopy (MRS) reveals significant alterations in levels of brain metabolites in the diabetic brain, which are consistent with and related to specific diabetic complications. Changes in these metabolites has been hypothesized to cause reduced neurotransmission, demyelination, neurodegeneration and brain atrophy. The study of brain metabolites and use of MRS in diabetic brain disease is at the initial stages. Further research and development could unveil the exact mechanism of diabetic brain injury as well as provide a new diagnostic tool to evaluate early disease and allow intervention [11].

In the case outlined above there are also nutritional factors which may have increased the risk of brain injury. Folic acid deficiency has been linked to cognitive impairment with behavioural disturbance in young adults [12]. Non-ketotic hyperglycaemia (NKH) can result in multiple neurological consequences such as seizures, hemichorea and hemianopia. In NKH, hyperglycaemia-induced blood brain barrier permeability contributes to epileptogenesis [13]. Therefore, we may hypothesize that recurrent hyperglycaemia and subsequent blood brain barrier permeability may have contributed to epileptogenesis and indeed brain injury in this case. Moreover, multiple sclerosis related blood-brain barrier permeability, although not the primary pathology in MS, may have contributed to reduced neuroprotection and increased risk of encephalopathy [14]. Factors such as nutritional deficiency, alcohol excess, cannabis use, depression and multiple sclerosis may have contributed to this patient’s susceptibility to encephalopathy however metabolic encephalopathy secondary to diabetic ketoacidosis was the most likely diagnosis.

In summary, metabolic encephalopathy is a devastating complication of DKA which can be due to both acute and chronic metabolic brain insults. To our knowledge this is the second case report describing this acute complication. It is an area of expanding research which will hopefully lead to improved understanding, treatment and patient outcome.

## Data Availability

None of the raw data pertaining to this case report is available publicly. The original investigation findings and reports are retained, as per normal procedure within the medical records of our institution.

## Abbreviations

AMPA: Alpha-amino-3-hydroxy-5-methyl-4-isoxazolepropionic acid
CSF: Cerebrospinal fluid
DKA: Diabetic Ketoacidosis
DNA: Deoxyribonucleic acid
ED: Emergency Department
EEG: Electroencephalogram
FLAIR: Fluid-attenuated inversion recovery
GABA: Gamma-aminobutyric acid
GAD: Glutamate acid decarboxylase
GCS: Glasgow Coma Scale
GFAP: Glial fibrillary acidic protein
H&E: Haematoxylin and eosin
HbA1c: Haemoglobin A1c
HBV: Hepatitis B virus
HCV: Hepatitis C virus
HHS: Hyperglycaemic Hyperosmolar Syndrome
HIV: Human Immunodeficiency Virus
HSV: Herpes Simplex Virus
IgG: Immunoglobulin G
IgM: Immunoglobulin M
MOG: Myelin Oligodendrocyte Glycoprotein
MRI: Magnetic Resonance Imaging
MRS: Magnetic resonance spectroscopy
NKH: Non-ketotic hyperglycaemia
NMDA: N-methyl-D-aspartate
NMO: Neuromyelitis optica
PCR: Polymerase Chain Reaction
POLG: DNA polymerase subunit gamma
RNP: Ribonuclear protein
RRMS: Relapsing Remitting Multiple Sclerosis
SGLT2: Sodium-Glucose-Co-Transporter 2
T1DM: Type 1 diabetes mellitus
TPO: Thyroid peroxidise
TTG: Tissue Transglutaminase
VGKC: Voltage gated potassium channel
